# Reducing Immunoreactivity of Gluten Peptides by Probiotic Lactic Acid Bacteria for Dietary Management of Gluten-Related Diseases

**DOI:** 10.3390/nu16070976

**Published:** 2024-03-27

**Authors:** Joanna Leszczyńska, Agnieszka K. Szczepankowska, Iwona Majak, Dorota Mańkowska, Beata Smolińska, Sylwia Ścieszka, Anna Diowksz, Bożena Cukrowska, Tamara Aleksandrzak-Piekarczyk

**Affiliations:** 1 Institute of Natural Products and Cosmetics, Faculty of Biotechnology and Food Sciences, Łódź University of Technology, Stefanowskiego 2/22, 90-530 Łódź, Poland; joanna.leszczynska@p.lodz.pl (J.L.); dorota.mankowska@p.lodz.pl (D.M.); beata.smolinska@p.lodz.pl (B.S.); 2 Institute of Biochemistry and Biophysics, Polish Academy of Sciences, Pawińskiego 5a, 02-106 Warsaw, Poland; agaszczep@ibb.waw.pl; 3 Institute of Technology and Food Analysis, Faculty of Biotechnology and Food Sciences, Łódź University of Technology, Stefanowskiego 2/22, 90-530 Łódź, Poland; iwona.majak@p.lodz.pl; 4 Institute of Fermentation Technology and Microbiology, Faculty of Biotechnology and Food Sciences, Łódź University of Technology, Wólczańska 171/173, 90-530 Łódź, Poland; sylwia.scieszka@p.lodz.pl (S.Ś.); anna.diowksz@p.lodz.pl (A.D.); 5 Immunology Laboratory, Department of Pathomorphology, The Children’s Memorial Health Institute, Dzieci Polskich 20, 04-760 Warsaw, Poland; b.cukrowska@ipczd.pl

**Keywords:** celiac disease, gluten-related diseases, gluten-free diet, endopeptidase, 33-mer peptide, peptidase-encoding genes, lactobacilli, probiotics

## Abstract

Immunoreactive gluten peptides that are not digested by peptidases produced by humans can trigger celiac disease, allergy and non-celiac gluten hypersensitivity. The aim of this study was to evaluate the ability of selected probiotic strains to hydrolyze immunoreactive gliadin peptides and to identify peptidase-encoding genes in the genomes of the most efficient strains. Residual gliadin immunoreactivity was measured after one- or two-step hydrolysis using commercial enzymes and bacterial peptidase preparations by G12 and R5 immunoenzymatic assays. Peptidase preparations from *Lacticaseibacillus casei* LC130, *Lacticaseibacillus paracasei* LPC100 and *Streptococcus thermophilus* ST250 strains significantly reduced the immunoreactivity of gliadin peptides, including 33-mer, and this effect was markedly higher when a mixture of these strains was used. In silico genome analyses of *L. casei* LC130 and *L. paracasei* LPC100 revealed the presence of genes encoding peptidases with the potential to hydrolyze bonds in proline-rich peptides. This suggests that *L. casei* LC130, *L. paracasei* LPC100 and *S. thermophilus* ST250, especially when used as a mixture, have the ability to hydrolyze immunoreactive gliadin peptides and could be administered to patients on a restricted gluten-free diet to help treat gluten-related diseases.

## 1. Introduction

Gluten is the general name for water-insoluble prolamin proteins of cereals, which include gliadin in wheat, secalin in rye and hordein in barley. Gluten can trigger gluten-related diseases such as celiac disease (CD), allergy and non-celiac gluten hypersensitivity [1]. The condition with the best understood pathomechanism associated with gluten intolerance is CD, which affects 0.7% of the world’s population. It is a chronic autoimmune enteropathy of the small intestine that occurs in individuals with a genetic predisposition manifested by the HLA-DQ2 and/or HLA-DQ8 haplotype [2].

Prolamines consist of multiple glutamine residues linked to prolines, making their structure highly complex and resistant to hydrolysis by proteolytic enzymes present in the human stomach and intestine [3]. Among gluten proteins, the α-gliadin fraction is the most immunopathogenic; after its digestion, the largest number of immunogenic peptides rich in proline and glutamine are obtained [4]. The 33-mer peptide from α2-gliadin (amino acid sequence positions 56–88, LQLQPFPQPQLPYPQPQLPYPQPQLPYPQPQPF) contains three overlapping T-cell epitopes (PQPQLPYPQ, PYPQPQLPY and PFPQPQLPY) that are thought to be crucial in the pathogenesis of CD [2,4,5]. Human gastrointestinal enzymes—pepsin, trypsin and chymotrypsin—are unable to hydrolyze the 33-mer peptide due to their inability to cleave before or after proline (P) or glutamine (Q), leaving the underlined epitopes intact. In CD, immunogenic gliadin peptides are translocated through the epithelial barrier to the mucosal lamina propria, where the intestinal enzyme tissue transglutaminase 2 (TTG2) converts glutamine residues to glutamic acid [2,6]. This conversion generates deamidated gluten peptides (DGPs), which strongly bind to HLA-DQ2/-DQ8 molecules on antigen-presenting cells. This activates specific T cells to induce pro-inflammatory responses and B cells to produce antibodies directed against TTG2 and DGPs [2].

The only available treatment for CD is a lifelong restrictive gluten-free diet (GFD). Unfortunately, patients are at risk of inadvertently ingesting gluten contained in medications, dietary supplements or certain foods, which can reduce the effectiveness of dietary treatment and evoke adverse health effects [7]. Therefore, various therapies are being researched to improve the health of CD patients and adherence to a GFD [7,8]. This includes a reduction in the immunoreactivity of gluten epitopes by introducing genetically, enzymatically or thermally modified wheat [9,10,11] or blocking zonulin, the protein, which may have implications for tight junctions’ sealing of the intestinal epithelium, and such an approach can reduce the absorption of gliadin peptides [12]. Blockers of HLA-DQ2/DQ8 molecules have also been studied to prevent the cascade of autoimmune reactions as well as TTG2 inhibitors that affect the formation of immunoreactive DPGs [8,13].

One of the most promising and also relatively easy methods of reducing gluten immunoreactivity is the use of probiotic lactic acid bacteria (LAB) strains with specific hydrolyzing properties. Such strains can be included in bread starter formulations [14] or applied as oral probiotics [15]. The main purpose of using probiotic bacteria for flour fermentation is to produce foods low in immunoreactive gluten peptides [14], while the oral administration of probiotics has multidirectional effects [16]. On the one hand, endopeptidase-containing bacterial strains may assist in the digestion of accidentally ingested gluten by CD patients; on the other hand, probiotics may have beneficial effects on modulating the composition of the gut microbiota, as well as activating anti-inflammatory immune processes in the intestine or strengthening the intestinal barrier [15,16]. Abnormalities in the composition of the intestinal microbiota have been described in CD patients (also in children at risk before the development of CD)—in general, the percentage of *Bacteroidetes* and *Proteobacteria* increases, while the content of *Lactobacillus*, *Bifidobacterium*, *Faecalibacterium prausnitzii* and *Streptococcus thermophilus* decreases [16,17]. Thus, orally administered probiotics could support dietary treatment by hydrolyzing immunoreactive peptides, as well as activating the health-promoting functions of the microbiome, which is also the case in other diseases [18]. However, it should be noted that the effect of probiotics is strain-dependent, therefore the need to find the most effective strain or a mixture of probiotics to achieve the digestion of immunoreactive gluten peptides and beneficial effects on the gut microbiome is emphasized [19]. 

The aim of the current study was to select probiotic strains with the highest ability to hydrolyze gliadin peptides, with particular focus on the hydrolysis of the immunoreactive 33-mer peptide. Unlike other studies that mainly examined probiotic strains from the genera *Lactobacillus* and *Bifidobacterium* [10,20], this study also evaluated other species such as *S. thermophilus* and *Weizmania coagulans*. In addition, most of the studies conducted to date have been based on demonstrating the ability to hydrolyze gliadin using various techniques for detecting immunoreactive peptides or isolating specific endopeptidases that degrade these peptides [10,11,16,20]. In contrast, in the current study, we pioneer a quantitative and qualitative link between the number of peptidases encoded in the genome and the presence of those specific for prolyl and glutamine bonds and the in vitro assessed ability to hydrolyze gliadin by selected probiotic strains. These results of a positive genome function correlation suggest that the identification of genes encoding peptidases with the potential to cleave proline and glutamine bonds in genomes could be the first selection of strains in the search for the most efficient scavengers of immunogenic peptides.

## 2. Materials and Methods

### 2.1. Probiotic Strains

This study was conducted using strains described in Table 1. All strains are deposited in the patent deposit of the Leibniz Institute DSMZ-German Collection of Microorganisms and Cell Cultures GmbH (Braunschweig, Germany) and were provided by Nordic Biotic Ltd. (Warsaw, Poland) and are part of the proprietary NORDBIOTIC^TM^ collection.

### 2.2. Obtaining Peptidase Preparations from Probiotic Strains

Peptidase preparations from probiotic strains were obtained according to a previously described method [24]. Sterile MRS medium (1800 mL) with 1% gluten (Sigma-Aldrich; St. Louis, MO, USA) was inoculated with a 12 h liquid culture of bacterial strains (10%) and grown without aeration (30–37 °C, 12 h). The overnight bacterial culture corresponding to 1 × 10^8^–1 × 10^9^ cells was separated by centrifugation (5000× *g*, 20 min, 5 °C) and washed twice with saline (20 mL, 0.9% NaCl) to obtain pelleted biomass. The biomass was then suspended in sterile saline solution (100 mL, 0.9% NaCl) and subjected to ultrasonic disintegration (30 min, 5 °C). The resulting homogenate was centrifuged to remove cell wall fragments (1000× *g*, 15 min, 5 °C), and the supernatant was taken as the bacterial lysate. To obtain peptidase preparations, bacterial lysates were frozen at −40 °C overnight, cooled for 1 h at −50 °C at atmospheric pressure, lyophilized at 20 °C for 24 h at 0.31 millibars and then dried at 30 °C at 0.0018 millibars for 1 h using a Chris delta 1–24 LSC freeze dryer (Teclen GmbH, Oberpframmern, Germany). Peptidase preparations were obtained in three biological repeats from independently grown bacterial cultures.

### 2.3. Two-Step Gliadin Hydrolysis Procedure

The evaluation of the ability of immunoreactive gliadin peptides to be hydrolyzed by peptidase preparations was carried out in a one- or two-step hydrolysis procedure according to previously described protocols [24,25]. As the initial step of the two-step hydrolysis procedure, the gliadin solution was hydrolyzed by pancreatin, a commercial mixture of digestive enzymes (lipase [10,000 units/150 mg pancreatin], amylase [8000 units/150 mg pancreatin] and protease [600 units/150 mg pancreatin]) provided in Kreon Travix (Mylan N.V.; Canonsburg, PA, USA) at a concentration of 0.045 g/mL in water, or by single enzymes: subtilisin (Sigma-Aldrich, USA), bromelain (Sigma-Aldrich, USA) or trypsin (Sigma-Aldrich, USA) at a concentration of 0.025 g/5 mL. The enzymatic reaction sample contained 5 mL of 0.2% gliadin solution in 30% ethanol, 5 mL of PBS buffer (pH 7.4) and 0.1 mL of solution of the respective enzymes. The enzymatic reaction was conducted for 2 h at 37 °C and terminated by incubating the samples in a boiling water bath for 10 min.

In the second step, 0.3 mL of peptidase preparation obtained individually from each strain was added at a concentration of 50 mg/5 mL to 1.2 mL of PBS buffer (pH 7.4) and to 0.5 mL of the hydrolysate prepared in the first hydrolysis step and incubated for 2 h at 37 °C. The untreated gliadin sample or gliadin prehydrolyzed by commercial enzymes subjected to an identical procedure without the addition of peptidase preparations served as a standard.

### 2.4. One-Step Gliadin Hydrolysis Procedure

During one-step hydrolysis, gliadin samples were simultaneously hydrolyzed with pancreatin (Kreon Travix; Mylan, USA), and individual peptidase preparations were obtained from single strains or in combination (where indicated). The following incubation mixture was used: 0.5 mL of 0.2% gliadin solution in 30% ethanol, 2.3 mL of PBS buffer pH 7.4, 0.1 mL of pancreatin at a concentration of 0.045 g/mL and 0.3 mL of a peptidase preparation from a single strain at a concentration of 50 mg/5 mL or a mixture of peptidase preparations in equal proportions [24]. The samples were incubated for 2 h at 37 °C. The untreated gliadin sample subjected to an identical procedure without the addition of peptidase preparations served as a standard.

### 2.5. Evaluation of Gliadin Content in Hydrolysates

In hydrolysates obtained by one- and two-step hydrolysis, gliadin content was assessed by immunoenzymatic assay with G12 (AgraQuant^®^ Gluten G12^®^; Romer Labs GmbH, Tulln an der Donau, Austria) and R5 (RIDASCREEN^®^ Gliadin; R-Biopharm Inc., Pfungstadt, Germany) antibodies according to the manufacturer’s instructions and previous report [5]. The G12 monoclonal antibody recognizes an immunoreactive 33-mer peptide, while the R5 monoclonal antibody recognizes a potentially immunogenetic QQPFP sequence that occurs multiple times in prolamine molecules. Assays were performed in triplicate, and gliadin content was calculated based on the standard curve. The results are presented as relative residual immunoreactivity [%] in respect to the untreated gliadin sample or prehydrolyzed gliadin by commercial digestive enzymes in the case of two-step hydrolysis.

### 2.6. Statistical and Bioinformatics Analyses

Data statistics were calculated using Excel 2007 (Microsoft Corp.; Redmond, WA, USA) and STATISTICA v. 10.0 (Dell Inc.; Round Rock, TX, USA) programs. The results were presented as arithmetic means with standard deviations. Comparisons between means were made using Tukey’s test for probability values *p* < 0.05. A one-way analysis of variance (ANOVA) with Tukey’s post hoc tests was performed to determine the significance of differences at *p* < 0.05. The normality of the distribution was checked using the Shapiro–Wilk test, and assumptions of homogeneity of variance were tested using the Brown–Forsythe test.

The cellular localization of the peptidases was assessed using the Protter tool [26]. The MEROPS database [27] was used to group peptidases into functional classes. Conserved domains (CDs) were identified using the NCBI Conserved Domain Database [28] and Pfam [29] search tools. Amino acid sequence similarity searches were performed using the Basic Local Alignment Search Tool (Blast) [30] with default parameter settings.

## 3. Results

### 3.1. The Impact of Two-Step Hydrolysis on the Immunoreactivity of Gliadin Peptides

**Figure 1:**
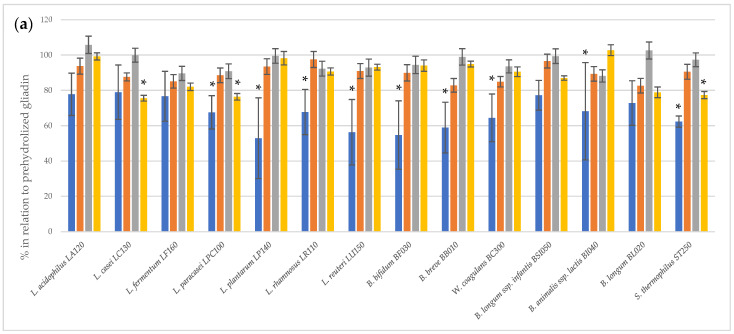

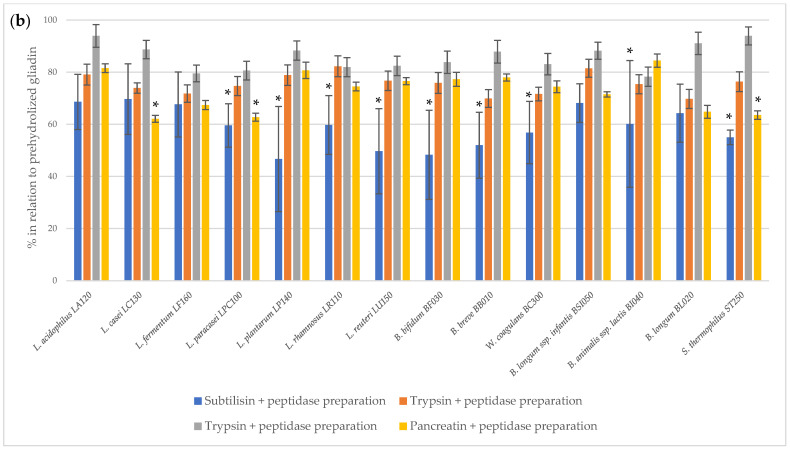
The relative residual gliadin immunoreactivity [%] after two-step hydrolysis assessed using the G12 test. (**a**), gliadin prehydrolyzed with commercial enzymes; (**b**), untreated gliadin. The results assess 33-mer immunoreactivity using the G12 test and are presented as means ± standard deviation from three independent biological repeats and two technical repetitions. Asterisks (*) mark statistical significance (*p* < 0.05).

**Figure 2:**
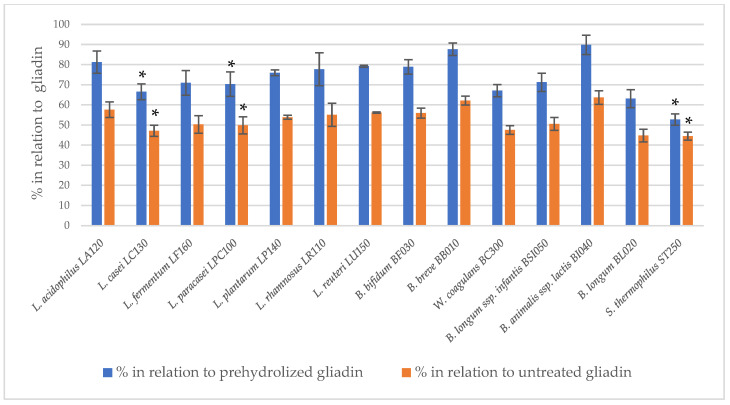
The relative residual gliadin immunoreactivity after prehydrolysis with pancreatin in the model of two-step hydrolysis assessed using the R5 test; the results assess gliadin peptide immunoreactivity using the R5 test and are presented as means ± standard deviation from three independent biological repeats and two technical repetitions. Asterisks (*) mark statistical significance (*p* < 0.05).

The immunoreactivity of peptides obtained after the two-step hydrolysis of gliadin was assessed using the 33-mer antibody-reactive test (G12 test) (Figure 1). The prehydrolysis of gliadin using bromelain and trypsin had no effect on the digestion performance of bacterial peptidase preparations. A statistically significant (*p* < 0.05) reduction in 33-mer residual immunoreactivity was observed following the action of bacterial peptidases after the initial hydrolysis of gliadin by subtilisin and pancreatin. For most of the tested bacterial peptidase preparations, a statistically significant reduction in 33-mer immunoreactivity was noted succeeding subtilisin digestion. In turn, peptidase preparations of only three strains, *L. casei* LC130, *L. paracasei* LPC100 and *S. thermophilus* ST250, were able to further reduce gliadin immunoreactivity after prehydrolysis with pancreatin. The R5 test confirmed that peptidase preparations from *L. casei* LC130, *L. paracasei* LPC100 and *S. thermophilus* ST250 strains significantly reduce the immunoreactivity of gliadin peptides other than 33-mer after initial hydrolysis with pancreatin (Figure 2), suggestive of the presence of gliadin-degrading peptidases in these specific bacterial preparations.

### 3.2. The Effect of the Mixture of Peptidase Preparations on Gliadin Hydrolysis

**Figure 3:**
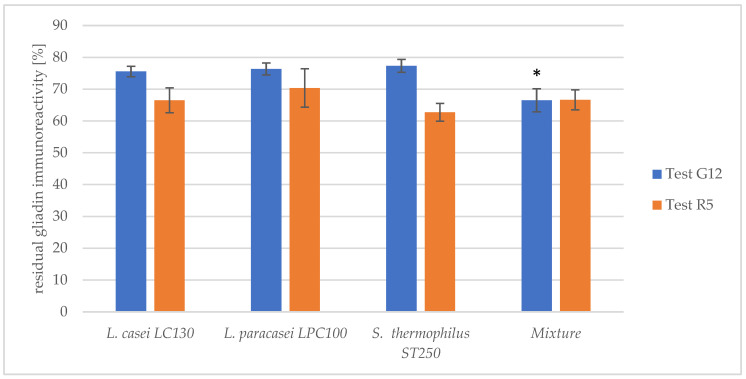
The residual gliadin immunoreactivity after treatment with the peptidase preparation from individual strains and their mixture in the one-step hydrolysis model with pancreatin. The results are presented as means ± standard deviation from three independent biological repeats and two technical repetitions and are shown as relative residual gliadin immunoreactivity in respect to untreated gliadin [%]. The assessment was conducted using the G12 and R5 tests. The asterisk (*) marks statistical significance (*p* < 0.05).

The synergistic impact of the most efficient peptidase preparations from strains *L. casei* LC130, *L. paracasei* LPC100 and *S. thermophilus* ST250 was investigated in a one-step hydrolysis model with pancreatin. A mixture of bacterial peptidase preparations from the three strains in the proportion 1:1:1 exhibited a significantly higher hydrolytic capacity towards 33-mer compared to peptidase preparations obtained from single strains (Figure 3). However, no reinforcing effect on the hydrolysis of gliadin peptides, as assessed with the R5 antibody, was observed after using the mixture. This suggested that the combination of the bacterial peptidase preparations is directed at hydrolyzing most particularly 33-mer rather than other gliadin peptides.

### 3.3. Genetic Potential of L. casei LC130 and L. paracasei LPC100 to Produce Peptidases

Genes encoding proteins with peptidase function were identified in the available genome sequences of *L. casei* LC130 and *L. paracasei* LPC100, which exhibited the highest potential for reducing gliadin immunoreactivity in the two-step hydrolysis model. For comparison, the same in silico analysis was performed on the available genome sequence of *L. plantarum* LP140, which showed one of the lowest potentials toward reducing gliadin immunoreactivity in the same model. A homology search for proteins encoded in the genomes of the three strains revealed the presence of many enzymes potentially involved in peptide hydrolysis, including those capable of cleaving peptide bonds formed by proline residues. However, individual strains differed in the number of encoded peptidases, with the highest quantity in *L. casei* LC130 and *L. paracasei* LPC100, 23 and 27, respectively, and only 18 identified in *L. plantarum* LP140 (Table 2). In exception to non-specific dipeptidases, which are more abundant in *L. plantarum* LP140, this strain has fewer duplicated genes encoding proline iminopeptidase PepI and oligoendopeptidase PepF (three of each in LPC100 and LC130 versus two in LP140) and a neutral endopeptidase PepO (two of each in LPC100 and LC130 versus one in LP140). In addition, *L. plantarum* LP140 is deficient in genes encoding bacillolysin, pyrrolidone-carboxylate peptidase (Pcp) and the S9 (prolyl oligopeptidase; POP) family peptidase, while both *L. paracasei* LPC100 and *L. casei* LC130 carry one copy of each of these genes (Table 2).

According to the MEROPS database [27], the POP family of peptidases consists of four subfamilies with different specificities toward peptide hydrolysis. The POP peptidases identified in *L. paracasei* LPC100 and *L. casei* LC130 were subjected to a more detailed in silico analysis to predict their potential function. A comparison of the amino acid sequence of the two POP peptidases (locus_tags VOW57_09600 and VIN14_08785 in, respectively, LPC100 and LC130) indicated 81% identity and 90% similarity between them, with variations occurring mostly in the N-terminal part of the protein, while the C-terminal half was more conserved. Both proteins had similar lengths (598 and 660 aa for VOW57_09600 and VIN14_08785, respectively) and domain arrangements—C-terminally localized peptidase S9, prolyl oligopeptidase, catalytic domain (Pfam 00326) and homology to dipeptidyl aminopeptidase/acylaminoacyl peptidase (COG1506: DAP2).

## 4. Discussion

The recommended GFD used to treat individuals with CD is not always effective, primarily due to inadvertent and unintended dietary errors [7]. Therefore, there is a continuous search for new ways to support dietary therapy in CD patients [14,15]. One such approach may involve the use of probiotic bacteria, which have the ability to produce peptidases that digest gluten peptides. However, lactobacilli and *Bifidobacterium* spp. that are most commonly used as probiotics have not been reported to synthesize proteolytic enzymes capable of hydrolyzing large protein molecules [10]; so far, these bacteria have only been found to produce membrane-bound endopeptidases that hydrolyze smaller molecules—peptides. This was consistent with the current in silico analyses, which revealed that for the best performing strains, *L. casei* LC130 and *L. paracasei* LPC100, only one peptidase was identified as having an extracellular localization. That is why for effective gliadin degradation by bacterial peptidase preparations, it was necessary to apply a prehydrolysis procedure with commercial peptidases: subtilisin—an enzyme with broad specificity, trypsin (a serine—protease)—catalyzing the hydrolysis of bonds formed by the carboxyl groups of arginine and lysine, and another serine protease—bromelain, as well as pancreatin (a mixture of lipase, amylase and proteolytic enzymes simulating digestive enzymes of the human digestive tract). The obtained results showed that only the action of subtilisin and pancreatin led to the digestion of gliadin to peptides, which showed a significant decrease in the immunoreactivity of the 33-mer peptide after the action of bacterial peptidases. The best effect of bacterial peptidases was observed after prehydrolysis by subtilisin (the peptidases of most strains reduced the concentration of immunoreactive gliadin peptides), but the subtilisin used in the current study was an enzyme produced by *Bacillus subtilis*, a bacterium that is not present in the human intestine under physiological conditions. The high efficiency of gluten digestion by subtilisin has also been demonstrated by other researchers [31,32]. Interestingly, Wei et al. identified *Rothia aeria*, a Gram-positive natural colonizer of the oral cavity and upper digestive tract, which was able to degrade and detoxify gluten in both in vitro and mouse in vivo models [32]. In this case, the gluten-degrading enzyme produced by this species was identified as a member of the subtilisin family. 

The current study allowed us to select probiotic strains that significantly decreased the immunoreactivity of gliadin peptides after initial hydrolysis by pancreatin, i.e., a mixture of enzymes naturally occurring in the human digestive tract. Such an effect was demonstrated by *L. casei* LC130, *L. paracasei* LPC100 and *S. thermophilus* ST250. Peptidases that degrade immunoreactive gliadin peptides have already been identified in various ‘lactobacilli’ by other authors [10,20,24,33]. Mickowska et al. also reported on the potential detoxification of gliadin by selected lactobacilli strains and fungal proteases (flavourzyme) to produce low-gluten bread [34]. The novelty of the current study is the selection of strains in an in vitro model simulating the digestion of gliadin in the gastrointestinal tract (using pancreatin). To the authors’ knowledge, there are no studies demonstrating the ability of *S. thermophilus* to digest immunoreactive gliadin peptides, as well as the synergic effect of the mixture of three strains of probiotic LAB.

The significant reduction in gliadin immunoreactivity caused by the activity of *L. casei* LC130 and *L. paracasei* LPC100 is supported by in silico data which revealed the presence of genes encoding peptidases with the potential to hydrolyze bonds in proline-rich peptides. In these two strains, an increased number of genes encoding typical proline-specific enzymes was found. In addition, there were also genes for unique peptidases that were absent in the genome of the poorly gliadin-hydrolyzing *L. plantarum* LP140. Among them were a member of the S9 family of serine peptidases, prolyl oligopeptidase (POP) and pyrrolidone-carboxylate peptidase (Pcp), which were both identified only in *L. casei* LC130 and *L. paracasei* LPC100, but not in the inefficient gliadin scavenger, *L. plantarum* LP140. Of these two unique enzymes, Pcp is involved in the removal of L-pyroglutamic acid from the amino-terminus of pyroglutamyl proteins or peptides [35] and has never been shown to have a role in the hydrolysis of gliadins, while POP endopeptidase is considered a key microbial enzyme in the degradation of immunogenic peptides [36,37,38]. Among the enzymes that were encoded in higher copy numbers in *L. casei* LC130 and *L. paracasei* LPC100 compared to *L. plantarum* LP140 were two metalloendopeptidases PepO and PepF and proline iminopeptidase PepI. All three peptidases are involved in the cleavage of prolyl bonds, and their important role in the digestion of gliadins has been previously indicated [36,39], suggesting that these enzymes may also be responsible for the increased peptide hydrolysis potential of *L. casei* LC130 and *L. paracasei* LPC100 observed in this study. Thus, the results of genomic analyses compiled with bacterial activities toward reducing gliadin immunogenicity suggest that the efficient bacterial hydrolysis of proline-rich peptides is due to an increased number of peptidases with the same activities and/or the presence of enzymes with specific properties. However, the confirmation of either version requires further research.

The oral administration of preclinically selected probiotic bacteria that support the digestion of immunoreactive peptides in the intestinal lumen could complement dietary treatment. It is also worth noting that in addition to the degradation of immunoreactive gliadin peptides, selected probiotic bacteria have a range of other health-promoting effects. The whole genomic sequences of *L. casei* LC130 and *L. paracasei* LPC100 were deposited in GenBank [40] and searched for genes with potential beneficial effects on human health. Four chromosomally encoded potential class IId bacteriocins were found that may contribute to the therapeutic effect of microbial dysbiosis in CD patients [16,17]. Complete pathways for efficient lactose metabolism have been identified, making *L. casei* LC130 and *L. paracasei* LPC100 efficient lactic acid producers with potent acidifying properties that can lower luminal pH and promote iron and calcium absorption [41,42]. Genes encoding enzymes of short-chain fatty acid synthesis pathways that may potentially fortify intestinal barrier function [43,44] have also been identified. In conclusion, current preclinical studies of selected probiotic strains indicate that a mixture of these strains can support the treatment of the GFD in patients with gluten-dependent diseases and have a positive impact on clinical syndromes such as anemia and bone mineralization disorders, which are currently the main extraintestinal manifestations of CD [45]. Therefore, it is planned to continue clinical trials in patients with gluten-dependent diseases to evaluate the effect of the orally administered probiotic strain mixture on gut microbiota composition and recovery during dietary treatment. A limitation of our study may be the lack of confirmation of the functionality of the identified in silico peptidase-encoding genes. Also, in vitro assays of gliadin content and immunoreactivity should eventually be confirmed by in vivo studies. However, due to the laboriousness and complexity of such analyses, this was not foreseen for the current work and is planned for upcoming studies.

## 5. Conclusions

Studies on peptidases derived from probiotic bacteria, particularly those targeting gliadins, are a relatively new area of research, and the efficacy and specificity of these enzymes in degrading immunoreactive gliadin fragments associated with CD are still under investigation. Here *L. casei* LC130, *L. paracasei* LPC100 and *S. thermophilus* ST250, especially when used as a mixture, have been presented to have the ability to hydrolyze immunoreactive gliadin peptides, which may mitigate their toxic effects in cases of the inadvertent consumption of gluten-containing foods or the accidental contamination of gluten-free foods by patients who should be on a restricted GFD and may help in the management of gluten-related diseases. An increased number of genes and the presence of unique genes encoding peptidases potentially involved in the hydrolysis of proline-rich peptides have been identified in the genomes of *L. casei* LC130 and *L. paracasei* LPC100. The enhanced hydrolysis potential of 33-mer peptides and other immunogenic peptides observed in vitro by these strains is likely due to the activity of their redundant peptidases. In this aspect, the availability of bacterial genome sequences facilitates the preliminary in silico screening of the number and catalytic type of encoded peptidases and, as a result, enables the selection of strains with the greatest potential to degrade immunogenic peptides. Anticipated future studies of the ability of specific peptidases to digest proline and glutamine bonds, as well as in vivo studies of the strains that most efficiently scavenge gliadin, should overcome the limitations of the in vitro and in silico studies conducted here and provide answers regarding the functionality of the systems analyzed.

## Figures and Tables

**Table 1:** Bacterial strains from the NORDBIOTIC^TM^ collection used in this study.

Species	Strain	DSMZ Number *
*Lactobacillus acidophilus*	LA120	33795
*Lacticaseibacillus casei* [Basonym: *Lactobacillus casei*]	LC130	33796
*Limosilactobacillus fermentum* [Basonym: *Lactobacillus fermentum*]	LF160	33805
*Lacticaseibacillus paracasei* [Basonym: *Lactobacillus paracasei*]	LPC100	33793
*Lactiplantibacillus plantarum* [Basonym: *Lactobacillus plantarum*]	LP140	33804
*Lacticaseibacillus rhamnosus* [Basonym: *Lactobacillus rhamnosus*]	LR110	33794
*Limosilactobacillus reuteri* [Basonym: *Lactobacillus reuteri*]	LU150	33841
*Bifidobacterium bifidum*	BF030	33818
*Bifidobacterium breve*	BB010	33814
*Weizmania coagulans/Heyndrickxia coagulans* [Basonym: *Bacillus coagulans*]	BC300	33836
*Bifidobacterium longum* ssp. *infantis* [Basonym: *Bifidobacterium infantis*]	BSI050	33813
*Bifidobacterium animalis* ssp. *lactis* [Basonym: *Bifidobacterium lactis*]	BI040	33812
*Bifidobacterium longum*	BL020	33815
*Streptococcus thermophilus*	ST250	33808

*, DSMZ—Leibniz Institute DSMZ-German Collection of Microorganisms and Cell Cultures GmbH; previously used species name is given in square brackets [21,22,23].

**Table 2:** Peptidase-encoding genes identified in silico in the chromosomes of *L. casei* LC130, *L. paracasei* LPC100 and *L. plantarum* LP140.

Enzyme Entry	General Classification	Locus_tag			Gene	Function	Cellular Location
		LPC100	LC130	LP140			
DIPETIDASES (EC 3.4.13)	Non-specific dipeptidases	VOW57_06340	VIN14_05430	V2P12_00955	*pepD*	Dipeptidase (EC 3.4.-.-)	cytoplasm
		VOW57_11255	VIN14_10245	V2P12_01380	*pepD*	Dipeptidase (EC 3.4.-.-)	cytoplasm
		VOW57_00205	VIN14_09190	V2P12_07345	*pepD*	Dipeptidase (EC 3.4.-.-)	cytoplasm
		VOW57_10190	-	V2P12_04350	*pepD*	Dipeptidase (EC 3.4.-.-)	cytoplasm
		VOW57_04325	-	V2P12_05760	*pepV*	Xaa-His dipeptidase (EC 3.4.13.-)	cytoplasm
	Proline-specific peptidases: proline dipeptidase (prolidase)	VOW57_04300	VIN14_03085	V2P12_09730	*pepQ*	Xaa-Pro dipeptidase (EC 3.4.13.9)	cytoplasm
METALLO ENDOPEPTIDASES (3.4.24)	Endopeptidases	VOW57_05660	VIN14_05080	V2P12_14540	*pepO*	Neutral endopeptidase O (EC 3.4.24.-)	cytoplasm
		VOW57_07925	VIN14_06965	-	*pepO*	Neutral endopeptidase O (EC 3.4.24.-)	cytoplasm
		VOW57_01280	VIN14_01015	V2P12_09460	*pepF*	Oligoendopeptidase F (EC 3.4.24.−)	cytoplasm
		VOW57_04015	VIN14_02800	V2P12_11255	*pepF*	Oligoendopeptidase F (EC 3.4.24.−)	cytoplasm
		VOW57_05320	VIN14_04820	-	*pepF*	Oligoendopeptidase F (EC 3.4.24.−)	cytoplasm
		VOW57_14970	VIN14_13985	-		Bacillolysin (EC 3.4.24.28)	extracellular
CYSTEINE PEPTIDASES (EC 3.4.22)	Major aminopeptidases: aminopeptidase C	VOW57_12010	VIN14_10970	V2P12_11255	*pepC*	Aminopeptidase C (EC 3.4.22.40)	cytoplasm
AMINOPEPTIDASES (EC 3.4.11)	Major aminopeptidases: aminopeptidase N	VOW57_02565	VIN14_01725	V2P12_04275	*pepN*	Lysyl aminopeptidase (EC 3.4.11.15)	cytoplasm
	Proline-specific peptidases: aminopeptidase P	VOW57_01540	VIN14_07915	V2P12_06815	*pepP*	Xaa-Pro aminopeptidase (EC:3.4.11.9)	cytoplasm
		VOW57_08765	-	-	*pepP*	Xaa-Pro aminopeptidase (EC:3.4.11.9)	cytoplasm
	Proline-specific peptidases: proline iminopeptidase	VOW57_04040	VIN14_02820	V2P12_00355	*pepI*	Proline iminopeptidase (EC 3.4.11.5)	cytoplasm
		VOW57_10215	VIN14_09220	V2P12_03920	*pepI*	Proline iminopeptidase (EC 3.4.11.5)	cytoplasm
		VOW57_13235	VIN14_12450	-	*pepI*	Proline iminopeptidase (EC 3.4.11.5)	cytoplasm
	Proline-specific peptidases: glutamyl (aspartyl) specific aminopeptidase	VOW57_01560	-	-	*pepA*	Glutamyl aminopeptidase (EC 3.4.11.7)	cytoplasm
	Specific aminopeptidases: methionine aminopeptidase	VOW57_05765	VIN14_05200	V2P12_01035	*pepM* (*map*)	Methionine aminopeptidase (EC 3.4.11.18)	cytoplasm
	Specific aminopeptidases: leucyl aminopeptidase	VOW57_05740	VIN14_05175	-	*pepS* (*ampS*)	Aminopeptidase S (EC 3.4.11.24)	cytoplasm
	Tripeptidases	VOW57_01570	-	V2P12_08125	*pepT*	Tripeptide aminopeptidase (EC 3.4.11.4)	cytoplasm
DI- and TRIPEPTIDYL PEPTIDASES (EC 3.4.14)	Proline-specific peptidases: X-prolyl-dipeptidyl aminopeptidase	VOW57_08805	VIN14_07955	V2P12_03935	*pepX*	Xaa-Pro dipeptidyl-peptidase (EC 3.4.14.11)	cytoplasm
OMEGAPEPTIDASES (EC 3.4.19)	Tripeptidases	-	VIN14_00705	-	-	γ-glutamyltranspeptidase (EC 2.3.2.2) @ Glutathione hydrolase (EC 3.4.19.13)	cytoplasm
	Specific aminopeptidases: pyrrolidone-carboxylate peptidase	VOW57_01075	VIN14_00785	-	*pcp*	Pyrrolidone-carboxylate peptidase (EC 3.4.19.3)	cytoplasm
SERINE PEPTIDASES	Proline-specific peptidases	VOW57_09600	VIN14_08785	-	*pop*	S9 (prolyl oligopeptidase, POP) family peptidase (EC 3.4.21.26)	cytoplasm
Unknown	Unspecified	VOW57_09535	VIN14_08720	V2P12_02345	-	Putative metallopeptidase (Zinc) SprT family	cytoplasm
Total genes		27	23	18			

## Data Availability

The data presented in this paper are available upon request from the corresponding author. The data are not publicly available for privacy reasons.
